# Molecular characteristics, risk factors, and clinical outcomes of methicillin-resistant *Staphylococcus aureus* infections among critically ill pediatric patients in Shanghai, 2016–2021

**DOI:** 10.3389/fped.2024.1457645

**Published:** 2024-10-17

**Authors:** Congyi Dai, Wenting Ji, Yufei Zhang, Weichun Huang, Haiying Wang, Xing Wang

**Affiliations:** ^1^ Department of Clinical Laboratory, Yueyang Hospital of Integrated Traditional Chinese and Western Medicine, Shanghai University of Traditional Chinese Medicine, Shanghai, China; ^2^ Department of Nursing, Huashan Hospital, Fudan University, Shanghai, China; ^3^ Department of Laboratory Medicine, Shanghai Children’s Medical Center, Shanghai Jiaotong University School of Medicine, Shanghai, China

**Keywords:** methicillin-resistant *staphylococcus aureus*, children, genotype, risk factors, mortality

## Abstract

**Objective:**

Methicillin-resistant *Staphylococcus aureus* (MRSA) infection in children has been on the rise, which poses a serious threat to their health and life in China. The purpose of this study was to determine the molecular characteristics, risk factors, and clinical outcomes of MRSA infections among critically ill pediatric patients.

**Methods:**

A retrospective case-control study was performed in the pediatric intensive care unit (PICU) of a tertiary university teaching hospital. All children infected with culture-positive *S. aureus* in the PICU between January 2016 and December 2021 were included. Univariate and multivariable logistic regression analyses were used to identify potential risk factors for MRSA infection and clinical outcomes of *S. aureus* infection. All *S. aureus* isolates were characterized based on antimicrobial resistance, multilocus sequence typing (MLST) and Staphylococcal protein A (*spa*) typing.

**Results:**

Of 3,974 patients admitted to the PICU, 280 were diagnosed with a *S. aureus* infection during the 6-year study period. Among them, 43.2% (121/280) were MRSA. All MRSA isolates showed significantly higher rates of resistance to penicillin, erythromycin, clindamycin and tetracycline than MSSA strains. The MRSA strains consisted of 45 *spa* types and 20 sequence types (STs) (20 clonal complexes), among which the most frequently represented were ST59-t437and ST398-t034. Multivariable logistic regression revealed vaginal delivery, respiratory failure, co-infection with a virus, C-reactive protein (CRP) > 8 mg/L as significant risk factors for MRSA infection. There was no significant difference in all-cause mortality during hospitalization between the MRSA group and the MSSA group. Furthermore, independent predictors for mortality in patients with *S. aureus* infections were the presence of hypoproteinemia, hematopathy, septic shock, respiratory failure, fever, and white blood cell (WBC) > 15.0 × 10^9^/L.

**Conclusions:**

The study revealed a high proportion of MRSA infections among critically ill pediatric patients, and found significant risk factors for MRSA infection and poor prognosis of *S. aureus* infection. Methicillin resistance did not contribute to the mortality in the current study. These findings will provide evidence-based practices to make the strategies of prevention and rational use of antibiotics for pediatric patients with *S. aureus* infection in the ICU.

## 1 Introduction

*S. aureus*, one of the most common Gram-positive pathogens, can trigger a variety of infectious diseases in children and adults, including community and hospital-acquired pneumonia, skin and soft tissue infections, infective endocarditis and bloodstream infections (1, 2). With the introduction of semisynthetic penicillins in 1959, methicillin-resistant *S. aureus* (MRSA) strains rapidly emerged in 1961 (3). Beginning in the 1980s, MRSA spread worldwide to such an extent that hospitals in many countries reported MRSA rates of 50% or more among *S. aureus* strains (4). Between January 1998 and June 2003, the annual average rate of MRSA in the United States increased to 51.6% in the ICU and 42% in non-ICU wards (5). In China, the prevalence of MRSA in children under the age of 18 still showed an increasing trend from 18.0% in 2005 to 29.8% in 2017 according to the China Antimicrobial Surveillance Network (CHINET) (6). The increase in the incidence of MRSA infection has become a serious problem in clinical fields, which is associated with increased morbidity and mortality, prolonged courses of antibiotics, extra length of hospital stay and excess hospital costs (7–9). In 2017, the World Health Organization (WHO) published a list of bacteria urgently needing new antimicrobial agents, MRSA was listed as a high-priority pathogen (10).

Genetically distinct MRSA lineages have been reported in most countries around the world, some of which were characterized by international epidemics, while others showed restricted geographic ranges (11, 12). For example, ST8 is mainly found in the United States, ST80 is prevalent in Europe, and ST59 is the most dominant ST in the Asia-Pacific region (11, 12). Moreover, the predominant MRSA lineage circulating in a country or region varies with time. Since about 2010, ST59 clone has gradually replaced ST239 as the dominant clone in most hospitals in China (13). In recent decades, there has been a growing body of research literature on MRSA infection in adults (14–16), but very little significant attention has been paid to the molecular characteristics and risk factors of MRSA infection in PICU patients. These patients tend to be critically ill which makes them extremely vulnerable to acquiring MRSA infections and less likely to survive (17).To bridge this gap, we launched a study to determine the molecular characteristics, risk factors, and clinical outcomes of MRSA infections among PICU patients from 2016 to 2021 in a large pediatric teaching hospital in Shanghai, China.

## 2 Materials and methods

### 2.1 Study design and population

The retrospective case-control study was performed at the PICU of Shanghai Children's Medical Center (SCMC) from January 2016 to December 2021. As one of the largest pediatric hospitals in Shanghai, SCMC is a teaching hospital affiliated to Shanghai Jiao Tong University, which is in charge of diagnosing, treating, and caring for newborns up to adolescents. The ethics committee of SCMC approved the study (SCMCIRB-K2023151-1) and granted to waive informed consent because of the nature of the retrospective study that minimizes risk to subjects.

During the study period, all children with culture-documented *S. aureus* infection in the PICU were identified through the Hospital Information System (HIS) and enrolled in this study. *S. aureus* infection was defined as isolation of *S. aureus* from at least one culture in addition to symptoms and signs compatible with inflammatory response. If more than one episode of *S. aureus* infection occurred in the same patient, only the first episode could be included.

To assess risk factors for death in patients with *S. aureus* infection, patients were divided into a survivor group and a non-survivor group. Survivors were defined as those who remained alive after discharge, and non-survivors as those who were died during hospitalization or discharged as terminally ill and refused treatment. A MRSA case was considered community-acquired if it was isolated from an inpatient within 48 h of hospitalization, and if risk factors for hospital-acquired infections, including recent (within 30 days) hospitalization or medical procedure (such as dialysis, surgery, and catheters), were absent (18).

### 2.2 Data collection

The following information was retrieved from electronic medical records and included in the database: demographic data (age, gender), information of birth (birth weight, premature, first child, multiple gestation, feeding style and delivery), comorbidities/underlying diseases, concomitant infection with other pathogens, clinical syndromes, medication and intervention therapy within 30 days preceding infection, history of hospitalization, length of hospital stay and PICU stay, clinical outcomes (length of hospital stay and PICU stay after infection, state of patients upon discharge, and hospital cost).

In addition, the following laboratory indicators were collected from the laboratory information system as well, such as C-reactive protein (CRP), white blood cell (WBC), platelet, hemoglobin, neutrophil/lymphocyte percentage, alanine transaminase (ALT), aspartate aminotransferase (AST), albumin (ALB), serum creatinine and serum urea.

### 2.3 Antimicrobial susceptibility testing

Bacterial species were firstly identified using an automated Vitek-2 system (bioMerieux, France) and further confirmed by the Vitek MS system (bioMerieux, France). All *S. aureus* isolates were tested for antibiotic susceptibility using the VITEK2 system with broth microdilution and disk diffusion methods. Sixteen antibiotics employed in the study were as follows: penicillin (P), oxacillin (OXA), cefoxitin (FOX), vancomycin (V), gentamicin (GM), erythromycin (E), tetracycline (TET), tigecycline (TGC), ciprofloxacin (CIP), levofloxacin (LEV), moxifloxacin (MOF), clindamycin (DA), rifampin (RIF), trimethoprim-sulfamethoxazole (SXT), quinupristin/dalfopristin (Q/D), and linezolid (LZD). Results were interpreted by the 2021 Clinical and Laboratory Standards Institute (CLSI) M100-S31 guidelines (19). *S. aureus* ATCC 29213 was used as the quality control strain.

Additionally, MRSA was defined as *S. aureus* that was resistant to oxacillin (minimum inhibitory concentration ≥ 4 µg/ml) or cefoxitin (MIC ≥ 8 µg/ml). Multidrug-resistant *S. aureus* (MDR-SA) was deﬁned as: (i) non-susceptibility to at least one agent in three or more antimicrobial categories, (ii) an MRSA is always considered MDR by virtue of being an MRSA (20).

### 2.4 Molecular typing

In order to understand the molecular characteristics of *S. aureus*, all isolates were characterized by multilocus sequence typing (MLST) and staphylococcal protein A (*spa*) typing. All *S. aureus* isolates were characterized according to the MLST protocol described by Enright MC et al. (21). Briefly, the chromosomal DNA of 280 *S. aureus* isolates was extracted by a standard phenol-chloroform extraction procedure. PCR amplicons of seven *S. aureus* housekeeping genes were obtained from chromosomal DNA. The PCR ampliﬁed products were sequenced (Sangon Biotech, Shanghai, China), and the sequences of the PCR products were assigned allele numbers by comparison with the existing sequences available on the MLST website for *S. aureus* (https://pubmlst.org/), the alleles of the seven genes deﬁned the *S. aureus* lineage, resulting in an allelic proﬁle designated ST. Clustering was based on STs related at the single-locus-variant level, which were deﬁned as clonal complexes (CCs), was determined using eBURST. *Spa* typing was determined by using the primers 1514R (CAG CAG TAG TGC CGT TTG CTT) and 1113F (TAA AGA CGA TCC TTC GGT GAG C) to amplify and sequence the polymorphic X-region of the *spa*, subsequently, *spa* types were assigned by using the *spa* database website (http://www.SpaServer.rindom.de).

### 2.5 Statistical analysis

Baseline and clinical characteristics of patients were summarized using frequencies and proportions for categorical variables, with comparisons performed using Fisher's exact test or Pearson's Chi-square test. For continuous variables exhibiting non-normal distributions, data were reported as medians and interquartile ranges (IQRs), with group differences assessed using the Mann-Whitney U test.

To investigate the associations between variables and the risk of MRSA infection and adverse outcomes of patients with *S. aureus* infection, we initially conducted regularized lasso regression with L1 regularization using the “glmnet” package in R to address multicollinearity and select relevant independent variables. Following this, stepwise logistic regression analysis was performed on the adjusted independent variables. Variance Inflation Factors (VIF) and tolerance values were calculated to ensure the absence of multicollinearity among the final predictors. Results of the risk factor analysis were reported as odds ratios (ORs) with 95% confidence intervals (CIs) and corresponding *p*-values. The significance level for all statistical analyses was set at *α* = 0.05. Correlation analyses were performed using SPSS version 26, while collinearity and logistic regression analyses were conducted using R version 4.4.1.

## 3 Results

### 3.1 Secular trend in the prevalence of MRSA infection in the PICU

**Figure 1:**
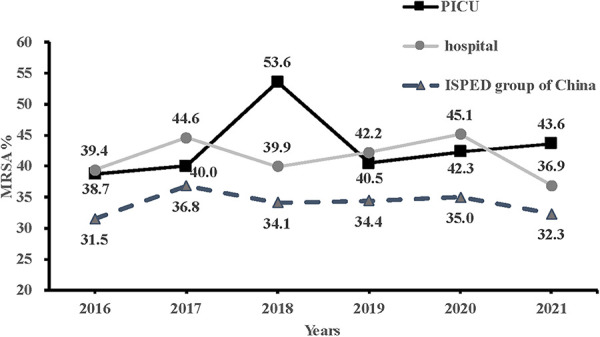
Comparison of trends in methicillin-resistant *Staphylococcus aureus* in the PICU, the entire hospital and in the ISPED group of China from 2016 to 2021. PICU, pediatric intensive care unit; ISPED, Chinese inspect survey of pediatric consortium.

Of the 3,974 PICU patients, 280 were diagnosed with *S. aureus* infections after admission to the PICU between January 2016 and December 2021. Among them, 43.2% (121/280) were MRSA, which was similar to the MRSA detection rate (41.4%, 1,004/2,425) in the entire hospital. As shown in Figure 1, the MRSA proportion in PICU showed a stable but slightly upward trend, from 38.7% in 2016 to 43.6% in 2021, whereas that in the entire hospital indicated a declining but fluctuating trend, from 39.4% in 2016 to 36.9% in 2021, which showed a similar tendency with MRSA proportion in group of China (22).

### 3.2 Antimicrobial susceptibility profiles among *S. aureus* isolates

In our study, 144(51.4%) were MDR strains, including all (100%) MRSA strains and 23 (14.5%) MSSA strains. Of 280 *S. aureus* isolates tested against 16 antibiotics, the rate of resistance to penicillin was the highest (91.8%), followed by erythromycin (52.9%), clindamycin (49.6%), oxacillin (43.2%) and tetracycline (20.0%). However, all isolates were uniformly susceptible to linezolid, vancomycin, tigecycline and quinupristin/dalfopristin. For the remaining antibiotics, the resistance rates were less than 10%.

As shown in Table 1, apart from oxacillin, resistance to rifampin was only observed in MRSA strains (1.7%). MRSA isolates showed significantly higher rates of resistance to penicillin (100% vs. 85.8%, *P* < 0.001), erythromycin (71.9% vs. 38.4%, *P* < 0.001), clindamycin (70.2% vs. 34.0%, *P* < 0.001), and tetracycline (25.6% vs. 15.7%, *P* = 0.040) than MSSA isolates.

**Table 1:** The antimicrobial resistance of *Staphylococcus aureus* strains in this study.

Antibiotics	Total *N* = 280	MSSA *N* = 159	MRSA *N* = 121	*P-*value
	*R* (*n*, %)	*R* (*n*, %)	*R* (*n*, %)	
Penicillin	257 (91.8)	136 (85.5)	121 (100)	**<0**.**001**
Erythromycin	148 (52.9)	61 (38.4)	87 (71.9)	**<0**.**001**
Clindamycin	139 (49.6)	54 (34.0)	85 (70.2)	**<0**.**001**
Oxacillin	121 (43.2)	0 (0.0)	121 (100)	**<0**.**001**
Tetracycline	56 (20.0)	25 (15.7)	31 (25.6)	**0**.**040**
Trimethoprim-sulfamethoxazole	23 (8.2)	15 (9.4)	8 (6.6)	0.394
Ciprofloxacin	14 (5.0)	6 (3.8)	8 (6.6)	0.280
Levofloxacin	13 (4.6)	5 (3.1)	8 (6.6)	0.172
Moxifloxacin	10 (3.6)	3 (1.9)	7 (5.8)	0.107
Gentamicin	6 (2.1)	3 (1.9)	3 (2.5)	1.000
Rifampicin	2 (0.7)	0 (0.0)	2 (1.7)	0.186
Linezolid	0 (0.0)	0 (0.0)	0 (0.0)	–
Vancomycin	0 (0.0)	0 (0.0)	0 (0.0)	–
Tigecycline	0 (0.0)	0 (0.0)	0 (0.0)	–
Quinupristin/dalfopristin	0 (0.0)	0 (0.0)	0 (0.0)	–

MRSA, methicillin-resistant *Staphylococcus aureus*; MSSA, methicillin-susceptible *Staphylococcus aureus*.

Bold values indicate significant *P* values <0.05.

### 3.3 Clinical and demographic characteristics of enrolled patients with *S. aureus* infection

During the six-year study period, all 121 children with MRSA infection in the PICU were classified as the case group, while all 159 children who suffered from MSSA infection were served as the control group. Ultimately, 280 pediatric inpatients who met the inclusion criteria were enrolled in the current study. From the clinical medical records, respiratory infection was the most frequently determined infection type. Among the specimens from which the 280 isolates were recovered, respiratory specimens accounted for 86.7% (243/280), including sputum (239, 85.4%), brochoalveolar lavage fluid (4, 1.4%), followed by blood (28, 10.0%), urine (3,1.1%), ocular and wound secretion (3, 1.1%), pleural fluid (2, 0.7%), and peritoneal fluid (1, 0.4%).

Demographic and clinical characteristics of children associated with *S. aureus* infection in the PICU are shown in Table 2. Of 280 study patients, 163 (58.2%) patients were male (Male/Female:1.4) and the median age was 0.5 years [interquartile range (IQR), 0.2–2.3 years]. One hundred and thirty-one (46.8%) were firstborn children, and the median birth weight reached 3.2 kg (IQR, 2.8–3.5 kg). 137 (48.9%) delivered vaginally and 162 (57.9%) began breastfeeding after birth.

**Table 2:** Comparison of clinical and epidemiological characteristics between MRSA and MSSA group based on univariate analysis.

Variable	Total	MSSA	MRSA	*P*-value
	*N* = 280	*N* = 159	*N* = 121	
Demographic information, *n* (%)				
Male sex	163 (58.2)	85 (53.5)	78 (64.5)	0.064
Age (years), median (IQR)	0.5 (0.2–2.3)	0.4 (0.2–1.5)	0.6 (0.2–3.0)	**0**.**031**
Birth weight (Kg), median (IQR)	3.2 (2.8–3.5)	3.3 (2.8–3.5)	3.2 (2.8–3.5)	0.508
First child	131 (46.8)	82 (51.6)	49 (40.5)	0.066
Multiple gestation	14 (5.0)	7 (4.4)	7 (5.8)	0.599
Vaginal delivery	137 (48.9)	69 (43.4)	68 (56.2)	**0**.**034**
Premature	35 (12.5)	21 (13.2)	14 (11.6)	0.682
Breastfeeding	162 (57.9)	87 (54.7)	75 (62.0)	0.223
Clinical symptoms, *n* (%)				
Fever	189 (67.5)	103 (64.8)	86 (71.1)	0.265
Fever (>39℃)	65 (23.2)	37 (23.3)	28 (23.1)	0.980
Persistent fever	57 (20.4)	33 (20.8)	24 (19.8)	0.850
Icterus	22 (7.9)	12 (7.5)	10 (8.3)	0.825
Diarrhea	19 (6.8)	9 (5.7)	10 (8.3)	0.391
Vomiting	15 (5.4)	11 (6.9)	4 (3.3)	0.184
Laboratory indicators, *n* (%)				
CRP > 8 mg/L	117 (41.8)	53 (33.3)	64 (52.9)	**0**.**001**
WBC > 15.0 × 10^9^/L	62 (22.1)	31 (19.5)	31 (25.6)	0.222
Platelet < 100 × 10^9^/L	38 (13.6)	18 (11.3)	20 (16.5)	0.207
Hemoglobin < 110 g/L	169 (60.4)	91 (57.2)	78 (64.5)	0.220
Neutrophil% > 70%	82 (29.3)	39 (24.5)	43 (35.5)	**0**.**045**
Neutrophil% < 50%	125 (44.6)	76 (47.8)	49 (40.5)	0.223
Lymphocyte% > 40%	122 (43.6)	73 (45.9)	49 (40.5)	0.365
Lymphocyte% < 20%	77 (27.5)	35 (22.0)	42 (34.7)	**0**.**018**
Albumin < 35 g/L	136 (48.6)	71 (44.7)	65 (53.7)	0.133
AST > 46U/L	122 (43.6)	69 (43.4)	53 (43.8)	0.946
ALT > 69U/L	45 (16.1)	27 (17.0)	18 (14.9)	0.635
CREA > 88 umol/L	3 (1.1)	2 (1.3)	1 (0.8)	1.000
UREA > 7.1 mmol/l	31 (11.1)	16 (10.1)	15 (12.4)	0.538
Pathogen co-infections, *n* (%)				
*Acinetobacter baumannii*	20 (7.1)	6 (3.8)	14 (11.6)	**0**.**012**
*Klebsiella pneumoniae*	14 (5.0)	6 (3.8)	8 (6.6)	0.280
*Escherichia coli*	11 (3.9)	6 (3.8)	5 (4.1)	1.000
*Pseudomonas aeruginosa*	7 (2.5)	3 (1.9)	4 (3.3)	0.470
Mycoplasma pneumoniae	13 (4.6)	5 (3.1)	8 (6.6)	0.172
Virus	50 (17.9)	17 (10.7)	33 (27.3)	**<0**.**001**
Comorbidities/underlying diseases, *n* (%)	241 (86.1)	134 (84.3)	107 (88.4)	0.320
Respiratory failure	142 (50.7)	66 (41.5)	76 (62.8)	**<0**.**001**
Severe pneumonia	128 (45.7)	69 (43.4)	59 (48.8)	0.372
Congenital heart disease	99 (35.4)	54 (34.0)	45 (37.2)	0.576
Anemia	52 (18.6)	31 (19.5)	21 (17.4)	0.648
Sepsis	44 (15.7)	24 (15.1)	20 (16.5)	0.744
Hematopathy	31 (11.1)	19 (11.9)	12 (9.9)	0.591
Hypoproteinemia	27 (9.6)	19 (11.9)	8 (6.6)	0.134
Malnutrition	25 (8.9)	11 (6.9)	14 (11.6)	0.176
Septic shock	21 (7.5)	14 (8.8)	7 (5.8)	0.342
Malignancy	9 (3.2)	5 (3.1)	4 (3.3)	1.000
Antibiotic exposure (within 30 days preceding infection), *n* (%)				
Two or more antibiotics	63 (22.5)	21 (13.2)	42 (34.7)	**<0**.**001**
3rd Cephalosporins	104 (37.1)	50 (31.4)	54 (44.6)	**0**.**024**
β-Lactam/β-Lactamase inhibitor combinations	37 (13.2)	11 (6.9)	26 (21.5)	**<0**.**001**
Carbapenems	23 (8.2)	8 (5.0)	15 (12.4)	**0**.**026**
Macrolides	23 (8.2)	6 (3.8)	17 (14.0)	**0**.**002**
Glycopeptides	22 (7.9)	9 (5.7)	13 (10.7)	0.117
Aminoglycosides	13 (4.6)	3 (1.9)	10 (8.3)	**0**.**012**
Antivirus-drug	14 (5.0)	3 (1.9)	11 (9.1)	**0**.**006**
Antifungal agents	12 (4.3)	4 (2.5)	8 (6.6)	0.094
Invasive operation (within 30 days preceding infection), *n* (%)	111 (39.6)	63 (39.6)	48 (39.7)	0.994
Endotracheal tube/Tracheostomy	73 (26.1)	39 (24.5)	34 (28.1)	0.500
Gastric intubation	69 (24.6)	44 (27.7)	25 (20.7)	0.177
Retention catheterization	30 (10.7)	16 (10.1)	14 (11.6)	0.686
Drainage-tube	27 (9.6)	16 (10.1)	11 (9.1)	0.785
Indwelling intravenous catheters	25 (8.9)	13 (8.2)	12 (9.9)	0.613
Bone marrow biopsy	17 (6.1)	8 (5.0)	9 (7.4)	0.404
Fiberoptic Bronchoscopy	16 (5.7)	9 (5.7)	7 (5.8)	0.964
Lumbar puncture	16 (5.7)	7 (4.4)	9 (7.4)	0.278
Treatments (within 30 days preceding infection), *n* (%)				
Immunosuppressive treatment^a^	94 (33.6)	54 (34.0)	40 (33.1)	0.874
Blood transfusion	74 (26.4)	45 (28.3)	29 (24.0)	0.415
Hydragogue	71 (25.4)	39 (24.5)	32 (26.4)	0.715
Intravenous immunoglobulin therapy	36 (12.9)	14 (8.8)	22 (18.2)	**0**.**020**
Surgery	27 (9.6)	15 (9.4)	12 (9.9)	0.892
Parenteral nutrition	14 (5.0)	6 (3.8)	8 (6.6)	0.280
Transplantation^b^ (within 1 year)	9 (3.2)	4 (2.5)	5 (4.1)	0.507
Origin of infection, *n* (%)				
Community-acquired	156 (55.7)	103 (64.8)	53 (43.8)	**<0**.**001**
Hospital-acquired	124 (44.3)	56 (35.2)	68 (56.2)	**<0**.**001**
History of hospitalization, *n* (%)	94 (33.6)	43 (27.0)	51 (42.1)	**0**.**008**

MRSA, methicillin-resistant *Staphylococcus aureus*; MSSA, methicillin-susceptible *Staphylococcus aureus*; IQR, interquartile range; CRP, C-reactive protein; WBC, white blood cell; AST, aspartate aminotransferase; ALT, alanine transaminase; CREA, creatinine; UREA, uric acid.

Bold values indicate significant *P* values < 0.05.

^a^ Immunosuppressive treatment defined as radiotherapy, chemotherapy, use of steroids and immunosuppressive agents.

^b^ Transplantation defined as solid organ transplantation or stem cell transplantation.

The most common clinical symptom was fever, which accounted for more than two-thirds of the study patients. Ninety-four (33.6%) cases had been previously hospitalized and up to 241 (86.1%) patients had at least one underlying disease, among which respiratory failure (142, 50.7%) was the most common, followed by severe pneumonia (128, 45.7%) and congenital heart disease (99, 35.4%). Approximately one-third of patients had received immunosuppressive treatment and 74 (26.4%) had undergone a blood transfusion in a month prior to infection. Furthermore, 22.5% received two or more antibiotics and the most widely used antibiotics were the third-generation cephalosporins. Additionally, 111 (39.6%) children were exposed to some sort of invasive procedure. Among them, tracheal intubation (73, 26.1%) and gastric intubation (69, 24.6%) were the most common.

### 3.4 Risk factors associated with MRSA infection among critically ill pediatric patients

The demographic and clinical characteristics of patients with MRSA and MSSA infection are summarized in Table 2. There was no difference in sex between the two groups. Compared to patients with MSSA infection, those suffered from MRSA infection tend to be older, have a higher proportion of vaginal delivery, respiratory failure, co-infection with *Acinetobacter baumannii* and virus. Three laboratory indicators, including CRP > 8 mg/L, neutrophil% > 70%, and lymphocyte% < 20%, also more frequently observed in patients with MRSA infection. Furthermore, more patients with MRSA infection received more than two antibiotics, the third-generation cephalosporins, β-lactam/β-lactamase inhibitor combinations, carbapenems, macrolides, aminoglycosides, and antivirus-drug (Table 2). Additionally, the MRSA group had longer hospital stays (*p* = 0.007), longer PICU stays (*P* = 0.013) and higher hospitalization expenses (*P* = 0.001) than the MSSA group (Table 3).

**Table 3:** Clinical outcomes of patients with *Staphylococcus aureus* infection.

Outcome	Total	MSSA	MRSA	*P*-value
	*N* = 280	*N* = 159	*N* = 121	
Length of hospital stay after *S. aureus* detection (days), median (IQR)	14 (7–27)	11 (6–24)	16 (8–31)	**0**.**007**
Length of PICU stay after *S. aureus* detection (days), median (IQR)	7 (3–14)	6 (3–12)	8 (4–16)	**0**.**013**
Hospital cost (yuan), median (IQR)	43,094.84 (15,116.86–102,135.11)	28,841.21 (12,420.04–86,794.03)	64,357.93 (18,859.26–142,275.75)	**0**.**001**
All-cause death during hospitalization	38 (13.6)	22 (13.8)	16 (13.2)	0.882

MRSA, methicillin-resistant *Staphylococcus aureus*; MSSA, methicillin-susceptible *Staphylococcus aureus*; IQR, interquartile range; PICU, pediatric intensive care unit.

Bold values indicate significant *P* values <0.05.

Multivariable logistic regression analysis revealed that vaginal delivery (*P* = 0.010), respiratory failure (*P* = 0.006), co-infection with a virus (*P* = 0.004) and CRP > 8 mg/L (*P* = 0.005) remained independent risk factors for acquiring MRSA infection (Table 4).

**Table 4:** Multivariable logistic regression analysis of risk factors for MRSA infection among critically ill pediatric patients.

Variable	*P* value	OR value	95% CI	VIF value	Tolerance
Vaginal delivery	0.010	2.034	1.182–3.498	1.060	0.943
Respiratory failure	0.006	2.162	1.260–3.726	1.070	0.934
Co-infection with a virus	0.004	2.839	1.405–5.734	1.017	0.984
CRP > 8 mg/L	0.005	2.167	1.252–3.734	1.043	0.959

MRSA, methicillin-resistant *Staphylococcus aureus*; PICU, pediatric intensive care unit; OR, odds ratio; CI, confidence interval; CRP, C-reactive protein; VIFs, variance inflation factors.

### 3.5 Clinical outcomes and risk factors of death of patients with *S. aureus* infection

During the hospital stay, 38 of 280 patients died, of which 25 patients discharged as terminally ill and refused treatment, the all-cause mortality rate was 13.6% in this study. The analysis of clinical outcomes and risk factors associated with mortality in patients with *S. aureus* infection is presented in Tables 5, 6, respectively. HA-MRSA infections occurred more frequently in the non-survival group (63.2% vs. 41.3%, *P* = 0.012), whereas CA-MRSA was more common in the survival group (36.8% vs. 58.7%, *P* = 0.012) (Table 5).

**Table 5:** Univariate analysis for factors associated with mortality of children with *Staphylococcus aureus* infections.

Variable	Total	Survival	No-survival	*P*-value
	*N* = 280	*N* = 242	*N* = 38	
Demographic information, *n* (%)				
Male sex	163 (58.2)	136 (56.2)	27 (71.1)	0.084
Age (years), Median (IQR)	0.5 (0.2–2.3)	0.4 (0.2–1.5)	2.9 (0.7–9.2)	**<0**.**001**
Birth weight (Kg), median (IQR)	3.2 (2.8–3.5)	3.2 (2.8–3.5)	3.3 (3.0–3.6)	0.108
First child	131 (46.8)	118 (48.8)	13 (34.2)	0.095
Multiple gestation	14 (5.0)	14 (5.8)	0 (0.0)	0.229
Vaginal delivery	137 (48.9)	119 (49.2)	18 (47.4)	0.836
Breastfeeding	162 (57.9)	138 (57.0)	24 (63.2)	0.477
Clinical symptoms, *n* (%)				
Fever	189 (67.5)	154 (63.6)	35 (92.1)	**<0**.**001**
Fever (>39℃)	65 (23.2)	50 (20.7)	15 (39.5)	**0**.**011**
Icterus	22 (7.9)	18 (74.)	4 (10.5)	0.516
Diarrhea	19 (6.8)	16 (6.6)	3 (7.9)	0.730
Vomiting	15 (5.4)	18 (7.4)	3 (7.9)	1.000
Laboratory indicators, *n* (%)				
CRP > 8 mg/L	117 (41.8)	93 (38.4)	24 (63.2)	**0**.**004**
WBC > 15.0 × 10^9^/L	62 (22.1)	48 (19.8)	14 (36.8)	**0**.**019**
Platelet < 100 × 10^9^/L	38 (13.6)	23 (9.5)	15 (39.5)	**<0**.**001**
Hemoglobin < 110 g/L	169 (60.4)	143 (59.1)	26 (68.4)	0.274
Neutrophil% > 70%	82 (29.3)	64 (26.4)	18 (47.4)	**0**.**008**
Neutrophil% < 50%	125 (44.6)	112 (46.3)	13 (34.2)	0.164
Lymphocyte% > 40%	122 (43.6)	111 (45.9)	11 (28.9)	0.051
Lymphocyte% < 20%	77 (27.5)	59 (24.4)	18 (47.4)	**0**.**003**
Albumin < 35 g/L	136 (48.6)	114 (33.3)	22 (57.9)	**0**.**003**
AST > 46U/L	122 (43.6)	103 (42.6)	19 (50.0)	0.390
ALT > 69U/L	45 (16.1)	37 (15.3)	8 (21.1)	0.368
CREA > 88 umol/L	3 (1.1)	0 (0.0)	3 (7.9)	**0**.**002**
UREA > 7.1 mmol/L	31 (11.1)	21 (8.7)	10 (26.3)	**0**.**004**
Pathogen co-infections, *n* (%)				
*Acinetobacter baumannii*	20 (7.1)	18 (7.4)	2 (5.3)	1.000
*Klebsiella pneumoniae*	14 (5.0)	10 (4.1)	4 (10.5)	0.106
*Escherichia coli*	11 (3.9)	9 (3.7)	2 (5.3)	0.649
*Pseudomonas aeruginosa*	7 (2.5)	5 (2.1)	2 (5.3)	0.243
Mycoplasma pneumoniae	13 (4.6)	12 (5.0)	1 (2.6)	1.000
Virus	50 (17.9)	42 (17.4)	8 (21.1)	0.580
Comorbidities/underlying diseases, *n* (%)	241 (86.1)	206 (85.1)	35 (92.1)	0.248
Respiratory failure	142 (50.7)	111 (45.9)	31 (81.6)	**<0**.**001**
Severe pneumonia	128 (45.7)	112 (46.3)	16 (42.1)	0.631
Congenital heart disease	99 (35.4)	92 (38.0)	7 (18.4)	**0**.**019**
Anemia	52 (18.6)	36 (14.9)	16 (42.1)	**<0**.**001**
Sepsis	44 (15.7)	32 (13.2)	12 (31.6)	**0**.**007**
Hematopathy	31 (11.1)	15 (6.2)	16 (42.1)	**<0**.**001**
Hypoproteinemia	27 (9.6)	16 (6.6)	11 (28.9)	**<0**.**001**
Malnutrition	25 (8.9)	20 (8.3)	5 (13.2)	0.355
Septic shock	21 (7.5)	8 (3.3)	13 (34.2)	**<0**.**001**
Malignancy	9 (3.2)	6 (2.5)	3 (7.9)	0.108
Antibiotic exposure (within 30 days preceding infection), *n* (%)				
Two or more antibiotics	63 (22.5)	52 (21.5)	11 (28.9)	0.306
3rd Cephalosporins	104 (37.1)	90 (37.2)	14 (36.8)	0.967
β-Lactam/β-Lactamase inhibitor combinations	37 (13.2)	32 (13.2)	5 (13.2)	1.000
Carbapenems	23 (8.2)	17 (7.0)	6 (15.8)	0.103
Macrolides	23 (8.2)	20 (8.3)	3 (7.9)	1.000
Glycopeptides	22 (7.9)	17 (7.0)	5 (13.2)	0.196
Aminoglycosides	13 (4.6)	10 (4.1)	3 (7.9)	0.395
Antivirus-drug	14 (5.0)	14 (5.8)	0 (0.0)	0.229
Antifungal agents	12 (4.3)	9 (3.7)	3 (7.9)	0.213
Invasive operation (within 30 days preceding infection), *n* (%)	111 (39.6)	63 (39.6)	48 (39.7)	0.994
Endotracheal tube/tracheostomy	73 (26.1)	57 (23.6)	16 (42.1)	**0**.**015**
Gastric intubation	69 (24.6)	55 (22.7)	14 (36.8)	0.061
Retention catheterization	30 (10.7)	22 (9.1)	8 (21.1)	**0**.**043**
Drainage-tube	27 (9.6)	24 (9.9)	3 (7.9)	1.000
Indwelling intravenous catheters	25 (8.9)	15 (6.2)	10 (26.3)	**<0**.**001**
Bone marrow biopsy	17 (6.1)	10 (4.1)	7 (18.4)	**0**.**003**
Fiberoptic Bronchoscopy	16 (5.7)	15 (6.2)	1 (2.6)	0.705
Lumbar puncture	16 (5.7)	9 (3.7)	7 (18.4)	**0**.**002**
Treatments (within 30 days preceding infection), *n* (%)				
Immunosuppressive treatment^a^	94 (33.6)	76 (31.4)	18 (47.4)	0.053
Blood transfusion	74 (26.4)	57 (23.6)	17 (44.7)	**0**.**006**
Hydragogue	71 (25.4)	55 (22.7)	16 (42.1)	**0**.**011**
Intravenous immunoglobulin therapy	36 (12.9)	27 (11.2)	9 (23.7)	0.063
Surgery	27 (9.6)	26 (10.7)	1 (2.6)	0.145
Parenteral nutrition	14 (5.0)	9 (3.7)	5 (13.2)	**0**.**028**
Transplantation^b^ (within 1 year)	9 (3.2)	8 (3.3)	1 (2.6)	1.000
Origin of infection, *n* (%)				
Community-acquired	156 (55.7)	142 (58.7)	14 (36.8)	**0**.**012**
Hospital-acquired	124 (44.3)	100 (41.3)	24 (63.2)	**0**.**012**
History of hospitalization, *n* (%)	94 (33.6)	78 (32.2)	16 (42.1)	0.231

MRSA, methicillin-resistant *Staphylococcus aureus*; MSSA, methicillin-susceptible *Staphylococcus aureus*; IQR, interquartile range; CRP, C-reactive protein; WBC, white blood cell; AST, aspartate aminotransferase; ALT, alanine transaminase; CREA, creatinine; UREA, uric acid; PICU, pediatric intensive care unit. Bold values indicate significant *P* values < 0.05.

^a^ Immunosuppressive treatment defined as radiotherapy, chemotherapy, use of steroids and immunosuppressive agents.

^b^ Transplantation defined as solid organ transplantation or stem cell transplantation.

**Table 6:** Multivariable logistic regression analysis of variables related to mortality of children with *Staphylococcus aureus* infection.

Variable	*P* values	OR values	95% CI	VIF values	Tolerance
Hypoproteinemia	0.004	4.71	1.633–13.582	1.016	0.985
Hemopathy	0.003	4.74	1.676–13.388	1.041	0.961
Septic shock	0.011	4.81	1.423–16.262	1.122	0.891
Respiratory failure	0.007	4.39	1.5.6–12.793	1.083	0.923
Fever	0.003	5.93	1.160–30.313	1.051	0.951
WBC > 150 × 10^9^/L	0.017	3.21	1.229–8.405	1.051	0.951

OR, odds ratio; CI, confidence interval; WBC, white blood cell; VIFs, variance inflation factors.

Compared with survivors, non-survivors had a higher proportion of fever, especially fever >39℃, CRP > 8 mg/L, WBC >15.0 × 10^9^/L, platelet <100 × 10^9^/L, neutrophil%  > 70%, lymphocyte% < 20%, albumin < 35 g/L, serum creatinine > 88 umol/L, and serum urea > 7.1 mmol/L. Moreover, some underlying diseases, including respiratory failure, anemia, sepsis, hematopathy, hypoproteinemia, and septic shock were more frequent in non-survivors. Besides, those who received specific treatments before *S. aureus* isolation, including endotracheal tube/tracheostomy, retention catheterization, indwelling intravenous catheters, bone marrow biopsy, lumbar puncture, blood transfusion, parenteral nutrition, and hydragogue (Table 5) were more likely to be non-survivors.

In the multivariable logistic regression model, independent risk factors associated with mortality in children with *S. aureus* infection included hypoproteinemia (*P* = 0.004), hepatopathy (*P* = 0.003), septic shock (*P* = 0.011), respiratory failure (*P* = 0.007), fever (*P* = 0.003) and WBC >15.0 × 10^9^/L (*P* = 0.017) (Table 6). There was no significant difference in the mortality between the MRSA group and the MSSA group (13.2% vs. 13.8%, *P* = 0.882) (Table 3).

### 3.6 Molecular characterization of *S. aureus* strains isolated from critically ill children

**Figure 2:**
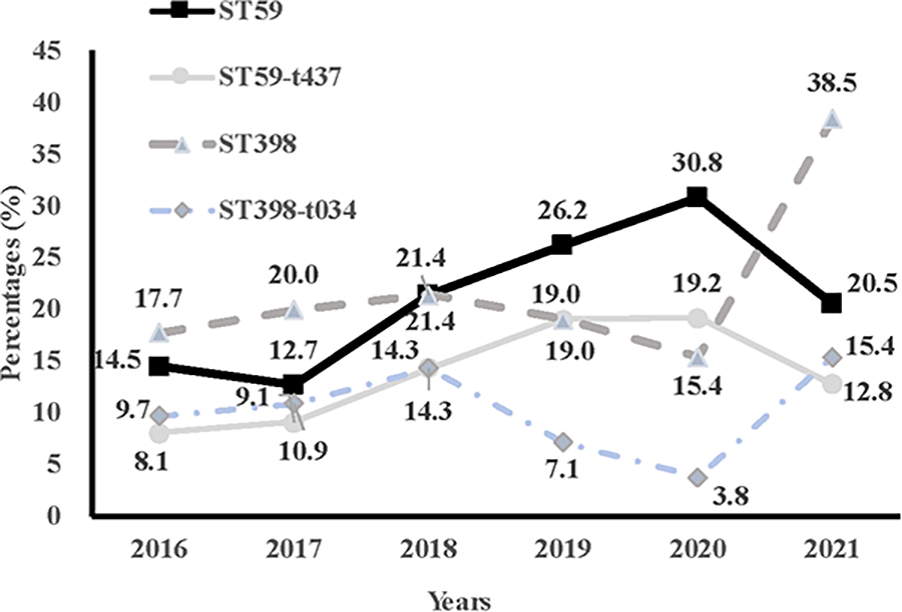
Comparison of trends of percentages of ST59, ST59-t437, ST398, and ST398-t034 in methicillin-resistant *Staphylococcus aureus* from 2016 to 2021.

The genetic diversity of all *S. aureus* isolates from children admitted to the PICU was analyzed by MLST and *spa* typing. There were 24 distinct sequence types (STs) identified within the 280 isolates, which belonged to 23 CCs (Table 7). CC398, CC59, CC22, CC25, and CC5 were the most prevalent CCs, including 21.8% (61/280), 19.6% (55/280), 9.6% (27/280), 8.5% (24/280), and 7.1% (20/280) of the isolated, respectively. While, the most frequently represented STs genotype was ST398 (21.8%, 61/280), followed by ST59 (19.6%, 55/280) and ST22 (9.6%, 27/280). The top three STs accounted for half of all *S. aureus* isolates. The *spa* typing discriminated 280 strains into 86 types, with t437 (12.9%, 36/280) as the most frequently represented type, followed by t034 (10.7%, 30/280), t309 (8.9%, 25/280), t571 (8.6%, 24/280) and t078 (7.1%, 20/280). Additionally, the percentage of ST59 genotype among the MRSA population, including the ST59-t437 genotype, showed an upward trend from 2016 to 2020 (except for 2021), whilst the proportion of ST398 genotype, including the ST398-t034 genotype, exhibited a wave-like uplift from 2016 to 2021 (Figure 2).

**Table 7:** Molecular characteristics of *Staphylococcus aureus* isolates from PICU patients.

MLST ST-CC	MRSA (*N* = 121)		MSSA (*N* = 159)	
	*n* (%)	*spa* type (*n*)	*n* (%)	*spa* type (*n*)
ST59-CC59	49 (40.5)	t437 (32), t172 (8), t441 (3), t1950 (1), t3517 (1), t3736 (1), t2906 (1), t3590 (1), t8886 (1)	6 (3.8)	t437 (4), t441 (1), t3527 (1)
ST398-CC398	30 (24.8)	t034 (23), t571 (3), t011 (1), t3275 (1), t5635 (1), t9266 (1)	31 (19.5)	t571 (21), t034 (7), t1250 (1), t1184 (1), t1456 (1)
ST5-CC5	9 (7.4)	t688 (4), t311 (3), t1473 (1), t2460 (1)	11 (6.9)	t548 (5), t002 (3), t5156 (1), t688 (1), t954 (1)
ST630-CC8	5 (4.1)	t4549 (4), t377 (1)	3 (1.9)	t377 (2), t4047 (1)
ST88-CC88	4 (3.3)	t5269 (1), t2310 (1), t3622 (1), t14777 (1)	1 (0.6)	t10777 (1)
ST26-CC25	3 (2.5)	t078 (3)	17 (10.7)	t078 (17)
ST25-CC25	1 (0.8)	t287 (1)	3 (1.9)	t349 (1), t280 (1), t258 (1)
ST121-CC121	3 (2.5)	t2092 (2), t2086 (1)	2 (1.3)	t8660 (1), t269 (1)
ST45-CC45	3 (2.5)	t015 (1), t026 (1), t116 (1)	1 (0.6)	t116 (1)
ST22-CC22	2 (1.7)	t309 (1), t3668 (1)	25 (15.7)	t309 (24), t5335 (1)
ST6-CC6	2 (1.7)	t304 (2)	6 (3.8)	t701 (5), t6797 (1)
ST72-CC72	2 (1.7)	t148 (1), t664 (1)	1 (0.6)	t148 (1)
ST15-CC15	1 (0.8)	t084 (1)	11 (6.9)	t084 (6), t085 (1), t346 (1), t360 (1), t279 (1), t803 (1)
ST188-CC188	1 (0.8)	t189 (1)	11 (6.9)	t189 (9), t8914 (1), t2883 (1)
ST30-CC30	1 (0.8)	t019 (1)	2 (1.3)	t4557 (1), t338 (1)
ST8-CC8	1 (0.8)	t9101 (1)	3 (1.9)	t9101 (2), t1705 (1)
ST1-CC1	1 (0.8)	t127 (1)	1 (0.6)	t401 (1)
ST239-CC8	1 (0.8)	t037 (1)	–	–
ST338-CC338	1 (0.8)	t3590 (1)	–	–
ST615-CC72	1 (0.8)	t324 (1)	1 (0.6)	t148 (1)
ST7-CC7	–	–	17 (10.7)	t091 (7), t796 (4), t14204 (2), t2883 (1), t605 (1), t9244 (1), t3338 (1)
ST20-CC20	–	–	4 (2.5)	t164 (2), t996 (1), t17523 (1)
ST1281-CC1281	–	–	1 (0.6)	t3076 (1)
ST3-CC3	–	–	1 (0.6)	t177 (1)

PICU, pediatric intensive care unit; ST, sequence type; MRSA, methicillin-resistant *Staphylococcus aureus*; MSSA, methicillin-susceptible *Staphylococcus aureus*.

Twenty distinct STs and 45 *spa* types were identified among MRSA strains, whereas 22 STs and 53 *spa* types were found with MSSA isolates. Among them, ST22-t309, ST398-t571, and ST26-t078 were the most common types of MSSA isolates, whereas ST59-t437 and ST398-t034 were overrepresented among MRSA isolates (OR = 13.933, *P* < 0.001, OR = 5.096, *P* < 0.001). Of note, ST398 was a unique genotype prevalent in both MRSA and MSSA. ST239 and ST338 genotypes were uniquely identified in MRSA strains, while ST7, ST20, ST1281 and ST3 were only found in MSSA strains. The remaining STs including ST398, ST59, ST22, ST26, ST5, ST15, ST188, ST6, ST630, ST121, ST88, ST25, ST45, ST8, ST30, ST72, ST1 and ST615 were found in both MRSA and MSSA.

Of the major prevalent strains, 89.1% of ST59 strains were MRSA, 49.2% of ST398 strains were MRSA and 7.4% of ST22 strains were MRSA. ST59 strains showed significantly higher rates of resistance to penicillin, erythromycin, clindamycin, and tetracycline than ST398 or ST22 strains (Supplementary Table S1). Except for methicillin resistance, ST398 and ST22 strains had no significant difference in resistance to other antibiotics.

## 4 Discussion

MRSA infections are a growing problem in pediatric patients, associated with high morbidity and mortality. In this retrospective study, we described the molecular characteristics, risk factors, and clinical outcomes of MRSA infections among critically ill pediatric patients between January 2016 and December 2021.

Clarifying resistance trends of MRSA is beneficial to guide clinical anti-infective treatment. In the United States, the incidence of MRSA bloodstream infections in hospitals and communities decreased by 74% and 40%, respectively, from 2006 to 2015 (23). In recent years, the prevalence of MRSA in the hospital has declined in some European countries, e.g., Austria, France, Ireland, the UK and Greece (24). In China, the proportions of MRSA among *S. aureus* infections in adults decreased from 69.94% in 2005 to 31.00% in 2020 (25, 26). In contrast, the prevalence of MRSA infections among critically ill pediatric patients found in our study remained in the range of 38.70%–43.5% during the six-year study period. These discrepancies may be attributed in part to the study population or the rise of community-associated MRSA (CA-MRSA) in children (27). Furthermore, all MRSA isolates showed a significantly higher rates of resistance to penicillin, erythromycin, clindamycin, and tetracycline than MSSA isolates. Consequently, MRSA infection should draw more attention in consideration of high drug resistance rate.

In recent years, there have been increasing studies on the molecular characteristics of MRSA infections in adults. However, the molecular profile of MRSA among Chinese children is limited. A study of *S. aureus* strains isolated from pediatric patients in Suzhou found that ST22 and ST59 were the most typical MRSA strains (28). Data from Guangzhou showed ST45 and ST59 were found to be major MRSA lineages among school-aged children across five schools (29). In contrast to these results, our results indicated that ST59 and ST398 were the dominant types of pediatric MRSA isolates. Among them, ST59-t437 and ST398-t034 genotypes are of particular concern, as they are 14 and 5 times more likely to cause MRSA infections in children, respectively. We observed the proportion of ST59 strain in all S. *aureus* strains increased year by year, mainly due to the increase in the proportion of MRSA-ST59-t437 infections. In comparison to an ST239-t030 strain, ST59-t437 MRSA lineage was reported to be characteristic of fast growth ability, high survival rate resistance, high toxin secretion levels, and cytotoxicity (30), which might facilitate its spread among pediatric patients, and maintain the most predominant MRSA clone in China (31). ST398 is a typical livestock-associated MRSA globally, which was first observed among pig and pig farmers in the Netherlands in 2003, and then found in Austria, Germany, and other countries (32). ST398-MRSA has been found more and more in humans and is associated with serious diseases. The virulence and biofilm formation of the ST398-MRSA subtype have been found to favor their adaptability in community and medical settings (33). Notably, we observed a further significant increase in the proportion of ST398 strain, from 17.7% in 2016 to 38.5% in 2021, especially the ST398-t034 lineage. Therefore, active surveillance is necessary to control and prevent the clinical impact of ST398-MRSA infections.

This study shed light on risk factors for MRSA infection in critically ill children. Patients with vaginal delivery, respiratory failure, co-infection with a virus, and CRP > 8 mg/L were significantly more likely to develop MRSA infection than MSSA infection. Pregnant women usually showed mild immunosuppression, elevated estrogen levels and blood glucose levels (34), which makes the MRSA colonization rate in pregnant women more than twice higher than in the general community (35). Furthermore, vaginal carriage represented a major risk factor for vertical transmission of this pathogen from mother to newborn (36), this may explain why pediatric patients with vaginal delivery are two times more likely to develop MRSA infections.

Our data revealed that respiratory failure is not only an independent risk factor for MRSA infection, but also for death due to *S. aureus* infection. Patients suffering from respiratory failure are often severely ill and tend to undergo invasive procedures such as mechanical ventilation support, which leads to an increased chance of bacterial infections, including MRSA (37). Moreover, severity of illness (such as Charlson complications score, septic shock and hematology tumor) has been significantly associated with poor prognosis in patients with *S. aureus* infection in many literatures (38, 39). It has even been reported that serious underlying diseases can lead to higher mortality rates regardless of the aetiologic agent (40). In addition, co-infection with a virus and high CRP value also increased the odds ratio for MRSA infection. Co-infection with a virus seems to be at an increased risk of severe diseases such as the development of respiratory failure, and patients would have a weaker immune system and are more vulnerable to MRSA infection. The high CRP values may be due in part to the fact that patients with MRSA infection have a higher proportion of co-viral infections and severe underlying diseases than those with MSSA infection.

In the current study, we found patients with MRSA infection had longer hospital stays and increased hospitalization costs than those with MSSA infection. However, no significant difference in all-cause mortality was observed between the two groups. In addition to microbial factors, host factors also play an important role in the progress of the disease (41). Serious underlying disease has ever been reported to be independently associated with mortality in patients with *S. aureus* infection (38, 39). Consistent with these studies, we found the presence of hypoproteinemia, hematopathy, septic shock and respiratory failure were independent predictors of worse outcomes for patients with *S. aureus* infection. Moreover, fever and WBC > 15.0 × 10^9^/L were another two predictors for mortality as well. WBC count was an inflammatory biomarker that reflected the underlying biological processes (42). Although it is not disease-specific, it is widely used to measure the severity of disease, tissue inflammation and infection.

There are several limitations in our study. Firstly, it was performed at a single center in China, and may not be generalizable to other institutions or settings. Secondly, there is no data exploring the virulence genes of *S. aureus* isolates, which would shed new light on the prognosis of patients with *S. aureus* infection. Thirdly, in our study, the *S. aureus* isolates were mainly recovered from sputum samples, accounting for 85.4%, which makes it more difficult to identify whether the pathogen is a colonized or an infected strain and easily leads to overestimate the detection rate of MRSA. Despite the above-mentioned limitations, this study spans a long time and recruited study patients through very strict inclusion and exclusion criteria, in order to minimize the selection bias, and strengthen the accuracy of data and validation of our results.

## 5 Conclusions

Through the 6-year study, a high proportion of MRSA infections were found in critically ill pediatric patients. These MRSA isolates consisted of 20 *spa* types and 45 sequence types (STs), among which the most frequently represented were ST59-t437 and ST398-t034. Multivariable logistic regression revealed vaginal delivery, respiratory failure, co-infection with a virus, C-reactive protein (CRP) > 8 mg/L as significant risk factors for MRSA infection. Methicillin resistance didn't contribute to the mortality associated with *S. aureus* infection in the current study. Furthermore, the presence of hypoproteinemia, hematopathy, septic shock, respiratory failure, fever, and WBC > 15.0 × 10^9^/L were independent predictors of mortality due to *S. aureus* infection. These findings will provide evidence-based practices to make the strategies of prevention and rational use of antibiotics for pediatric patients with *S. aureus* infection stay in the ICU.

## Data Availability

The original contributions presented in the study are included in the article/Supplementary Material, further inquiries can be directed to the corresponding authors.
