# Supplementary material for: Molecular characteristics, risk factors, and clinical outcomes of methicillin-resistant Staphylococcus aureus infections among critically ill pediatric patients in Shanghai, 2016–2021
Source: Front Pediatr. 2024 Oct 17;12:1457645. doi: 10.3389/fped.2024.1457645 (PMC11524809; doi:10.3389/fped.2024.1457645)
Supplement: Supplementary file 1 [file Table1.pdf]

Supplementary table 1 Antimicrobial susceptibility profiles among the molecular types of 280 *S. aureus* isolates.

MLST	Isolates	P[a]	OXA[a]	GM[a]	E[a]	TET[a]	CIP[a]	LEV[a]	MOF[a]	DA[a]	SXT[a]	RIF[a]
ST398	61	86.9	49.2	0	47.5	9.8	3.3	3.3	3.3	45.9	4.9	0
ST59	55	100	89.1	0	81.8	40	0	0	0	80	1.8	0
ST22	27	92.6	7.4	0	51.9	7.4	3.7	3.7	0	44.4	0	0
ST26	20	85	15	0	45	25	0	0	0	40	20	0
ST5	20	90	45	10	60	20	25	25	25	65	10	0
ST7	17	100	0	11.8	29.4	41.2	5.9	5.9	5.9	29.4	17.6	0
ST15	12	75	8.3	0	25	16.7	0	0	0	16.7	0	0
ST188	12	83.3	8.3	0	25	0	0	0	0	16.7	0	8.3
ST6	8	100	25	0	25	12.5	0	0	0	25	0	0
ST630	8	100	62.5	0	50	37.5	25	25	0	25	0	0
ST121	5	80	60	0	40	40	0	0	0	40	80	0
ST88	5	100	80	0	80	0	0	0	0	80	20	0
ST20	4	100	0	0	75	0	25	0	0	75	0	0
ST25	4	100	25	0	25	0	0	0	0	25	25	0
ST45	4	100	75	0	50	0	0	0	0	50	0	0
ST8	4	100	25	25	75	25	25	25	25	50	25	0
ST30	3	66.7	33.3	0	100	0	0	0	0	100	0	0
ST72	3	66.7	66.7	0	33.3	0	0	0	0	33.3	0	0
ST1	2	100	50	0	0	0	0	0	0	0	50	0
ST615	2	100	50	0	50	0	0	0	0	50	50	0
ST1281	1	100	0	0	0	0	0	0	0	0	0	0
ST239	1	100	100	0	100	100	100	100	0	100	100	100
ST3	1	100	0	0	0	0	0	0	0	0	0	0
ST338	1	100	100	100	100	0	0	0	100	100	0	0

Total	280	91.8	43.2	2.1	52.9	20	5	4.6	3.6	49.6	8.2	0.7
P-value	ST398 vs. ST59	**0.007**	**<0.001**	-	**<0.001**	**<0.001**	0.497	0.497	0.497	**<0.001**	0.62	-
P-value	ST398 vs. ST22	0.717	**<0.001**	-	0.709	1	1	1	1	0.899	0.55	-
P-value	ST59 vs. ST22	0.106	**<0.001**	-	**0.005**	**0.002**	0.329	0.329	-	**0.001**	0.329	-

[a]: P, penicillin; OXA, oxacillin; GM, gentamicin; E, erythromycin; TET, tetracycline; CIP, ciprofloxacin; LEV, levofloxacin; MOF, moxifloxacin; DA, clindamycin; SXT, trimethoprim-sulfamethoxazole; RIF, rifampin.
